# Single-incision totally extraperitoneal hernia repair with intraperitoneal inspection of strangulated femoral hernia at risk for intestinal ischemia after repositioning: a case report

**DOI:** 10.1186/s13256-019-2134-4

**Published:** 2019-07-16

**Authors:** Yosuke Namba, Takashi Urushihara, Hideki Nakahara, Toshiyuki Itamoto

**Affiliations:** 1 Department of Gastroenterological Surgery, Hiroshima Prefectural Hospital, 1-5-54 Ujina-kanda, Minami-ku, Hiroshima, 734-8530 Japan; 2 Department of Gastroenterological and Transplant Surgery Applied Life Sciences, Institute of Biomedical and Health Sciences, Hiroshima University, Hiroshima, Japan

**Keywords:** Femoral hernia, Single incision, Totally extraperitoneal hernia repair

## Abstract

**Background:**

Totally extraperitoneal hernia repair and the transabdominal preperitoneal approach have advantages and disadvantages. We used the advantages of totally extraperitoneal hernia repair and the transabdominal preperitoneal approach and performed single-incision totally extraperitoneal hernia repair with intraperitoneal inspection for the treatment of strangulated femoral hernia in a patient at risk for intestinal ischemia.

**Case presentation:**

We report a case of a 75-year-old Japanese woman who presented with black vomiting of 5 days’ duration. Physical examination revealed a right inguinal bulge and sharp pain. Computed tomography revealed a right strangulated femoral hernia with no intestinal ischemia. We were able to reposition the hernia; however, we performed the operation with consideration of the possibility of intestinal ischemia by incarceration of the intestine because the onset was 5 days previously. Intraperitoneal observation revealed a right femoral hernia and confirmed that the intestinal tract was not ischemic. However, the intestinal tract was expanded because of ileus, and securing a clear field of vision was difficult. Hence, we switched to totally extraperitoneal hernia repair at the same incision and performed single-incision totally extraperitoneal hernia repair with intraperitoneal inspection. The hernia sac was observed at the femoral rings and obturator foramen. The mesh was inserted through the incision, and after it was positioned to cover the Hesselbach triangle, femoral rings, inguinal ring, and obturator foramen, it was fixed with SECURESTRAP®. Observation of the abdominal cavity confirmed complete repair. After the operation, there was no recurrence or other complications.

**Conclusion:**

We report a case of successful single-incision totally extraperitoneal hernia repair with intraperitoneal inspection for the treatment of strangulated femoral hernia in a patient at risk for intestinal ischemia after repositioning.

## Background

Surgical treatment of inguinal and femoral hernia has radically changed over the years. Recently, discussions on inguinal and femoral hernia repair have focused not only on the rate of recurrence but also on chronic pain [1, 2]. Laparoscopic repair is associated with less postoperative pain, faster return to normal activities, and less chronic pain than classic open, tension-free mesh repair [3]. Totally extraperitoneal hernia repair (TEP) and the transabdominal preperitoneal approach (TAPP) are effective methods of laparoscopic primary inguinal and femoral hernia repair. In TAPP, we can diagnose the type of hernia by intraperitoneal observation. However, covering the hernia with a big mesh is difficult, and the field of vision is limited by the intestinal tract. In TEP, the covering mesh and the field of vision are not influenced by the intestinal tract; however, intraperitoneal observation cannot be performed. In cases of intestinal incarceration, intraperitoneal observation may be necessary to confirm the presence of intestinal damage after reduction. Because the intestinal tract is expanded by the ileus, securing a clear field of vision is difficult. In this situation, TEP is useful for the surgical procedure. Hence, using the advantages of TEP and TAPP is important for the treatment of femoral hernia in patients at risk of intestinal ischemia and is complicated with ileus. Hence, we performed single-incision totally extraperitoneal hernia repair with intraperitoneal inspection (iSTEP) in our patient with this presentation [4].

## Case presentation

**Fig. 1:**
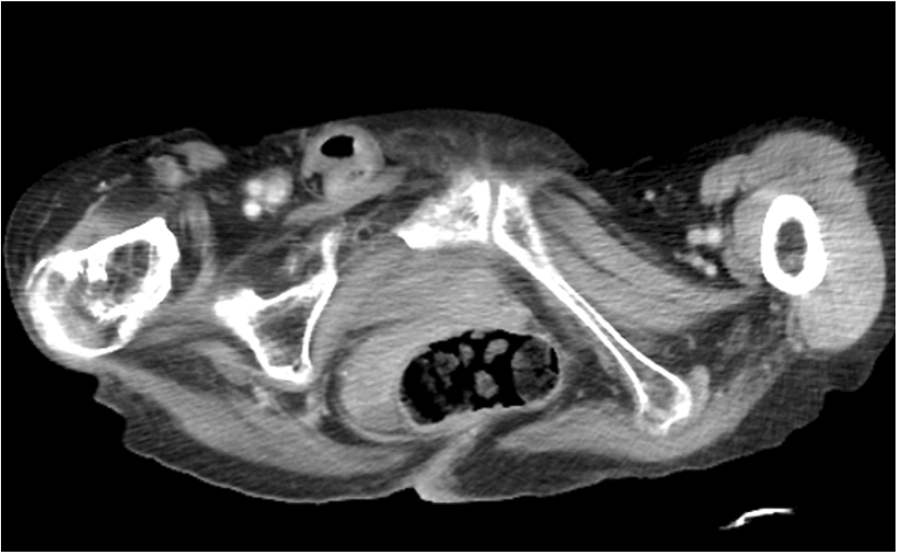
Computed tomography (CT). Enhanced CT shows a right strangulated femoral hernia. The intestinal wall shows enhancement

A 75-year-old Japanese woman with dementia and disuse syndrome presented with black vomiting. Her physical examination demonstrated a right inguinal bulge and sharp pain. Computed tomography revealed a right strangulated femoral hernia with no intestinal ischemia (Fig. 1). She had ileus complicated by incarcerated femoral hernia, which we repositioned. However, we performed the operation with consideration of the possibility of intestinal ischemia because the onset was 5 days previously. Laparoscopic intraperitoneal observation was initially done to check for intestinal nonischemia.

**Fig. 2:**
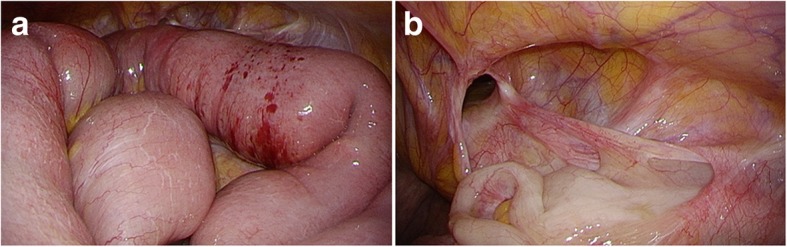
Intraperitoneal observations. **a** The intestinal tract was not ischemic. However, the intestinal tract was partially reddish and expanded. **b** The patient had a right femoral hernia

**Fig. 3:**
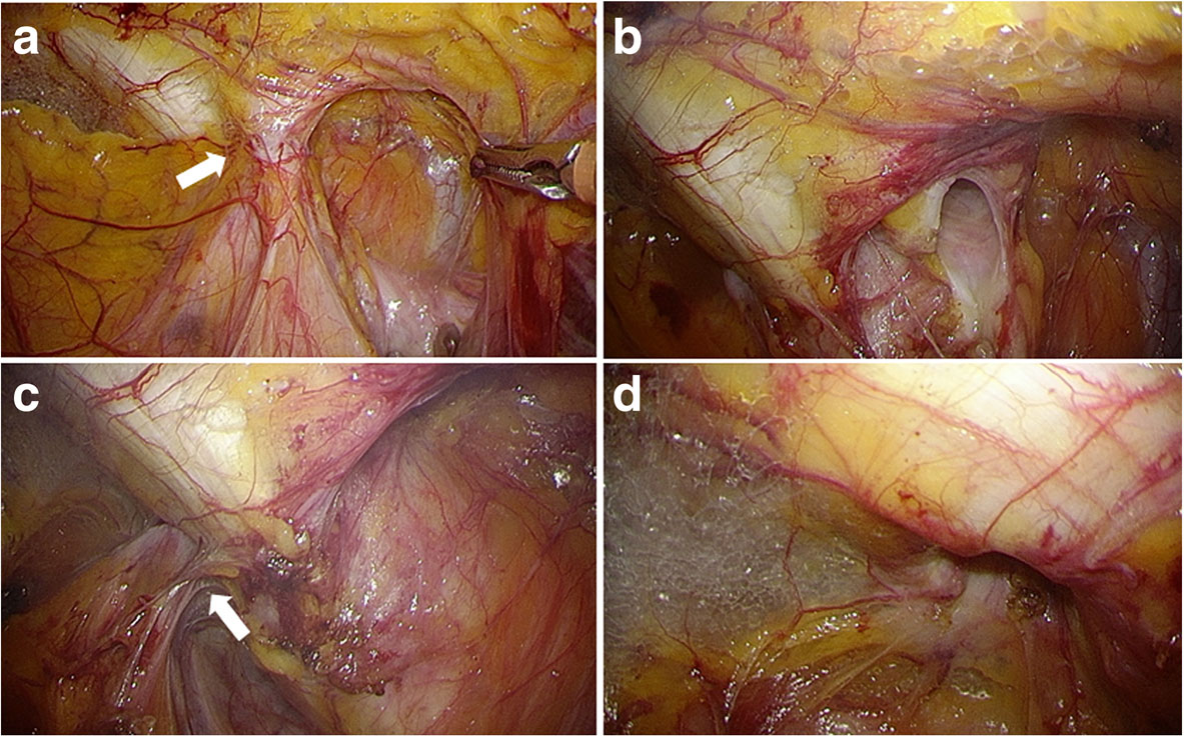
Preperitoneal space. **a** The hernia sac was observed at the femoral orifice (arrow). **b** The femoral hernia sac was dissected and fully withdrawn into the peritoneal cavity, and the femoral ring was confirmed. **c** We found the hernia sac at the obturator foramen (arrow). **d** The right obturator hernia sac was dissected and withdrawn into the peritoneal cavity

**Fig. 4:**
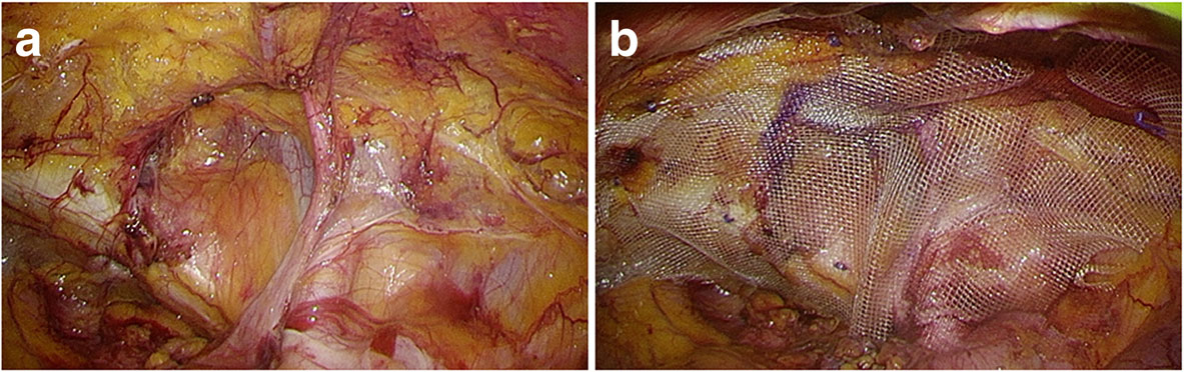
Right inguinal floor. **a** Complete dissection of the right inguinal floor with identification of Cooper’s ligament, inferior epigastric vessels, and round ligament. **b** A TiLENE mesh was positioned to cover the Hesselbach triangle, femoral rings, inguinal ring, and obturator foramen, and it was fixed using a secure strap

**Fig. 5:**
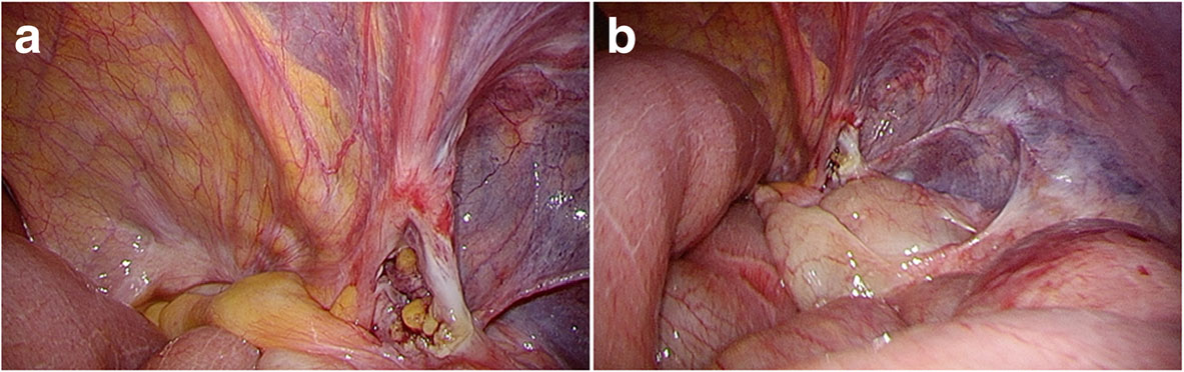
Observation of the abdominal cavity revealed that the repair was complete. **a** We reconfirmed the repair of the femoral region. **b** We confirmed the mesh covering with the internal inguinal ring

During the laparoscopic surgery, the patient was placed in supine position under general anesthesia. A 2-cm transverse skin incision was made in the umbilicus, followed by an incision in the peritoneum from the fascia defect to the abdominal cavity. A 10-mm trocar attached to an access port was inserted, and carbon dioxide was insufflated at 8 mmHg. We diagnosed a right femoral hernia and confirmed that the intestinal tract was not ischemic (Fig. 2a, b). However, the intestinal tract was expanded because of ileus, and securing a clear field of vision was difficult. Hence, we switched to TEP at the same incision and performed iSTEP. The trocar was removed, and the peritoneum was closed after a catheter was inserted to degas the cavity. The peritoneum was closed and ligated with 3-0 Vicryl (Ethicon, Somerville, NJ, USA). The rectus abdominis was split, and the posterior sheath was exposed. A multichannel access port (GelPOINT MINI; Applied Medical, Rancho Santa Margarita, CA, USA) was installed in the preperitoneal space, and carbon dioxide was insufflated at 8 mmHg. The preperitoneal space was dissected using a bipolar forceps by pulling toward the Retzius cavity, and the peritoneal edge was checked. The hernia sac was observed at the femoral rings, confirming the diagnosis of femoral hernia (Fig. 3a, b). The peritoneal edge was grasped and dissected toward the dorsal and lateral sides to secure a space for the mesh. We also found a part of the hernia sac at the obturator foramen and secured a space for mesh equally (Fig. 3c, d). A 10 × 15-cm TiLENE mesh (PFM Medical, Cologne, Germany) was inserted through the incision. After the mesh was positioned to cover the Hesselbach triangle, femoral rings, inguinal ring, and obturator foramen, it was fixed to the Cooper’s ligaments, interior side, and lateral sides using SECURESTRAP® (Ethicon Endosurgery, Cincinnati, OH, USA) (Fig. 4a, b). Observation of the abdominal cavity revealed that the repair was complete (Fig. 5a, b). The total procedure time was 49 minutes, and blood loss was 1 ml. After undergoing treatment of the paralytic ileus and undergoing rehabilitation, the patient was transferred to another hospital and had no recurrence or other complications.

## Discussion

Compared with the conventional anterior approach, laparoendoscopic repair results in less postoperative pain, fewer postoperative complications, lower recurrence rates, early discharge, and faster return to normal daily activities [5]. However, Köckerling *et al.* reported that univariable and multivariable analyses did not reveal any significant difference between TEP and TAPP with regard to intraoperative and perioperative complications [6]. TEP and TAPP have advantages and disadvantages, and it is important to use TEP and TAPP properly based on individual cases. In our patient, treatment with only TEP or TAPP was difficult.

We compared the advantages and disadvantages of each procedure. In TAPP, intraperitoneal observation can diagnose the type of hernia and confirm repair after the application of the covering mesh. However, covering with a proper big mesh and dissecting the abdominal wall side are difficult, and the field of vision is limited if the intestinal tract has adhesion. Additionally, Gass *et al.* reported that the postoperative length of hospital stay after TAPP was longer than that of TEP [7]. Meanwhile, wide dissection is possible in TEP; furthermore, application of the covering mesh and dissection of the abdominal wall side are easy. Some studies have reported lower pain in TEP, because the mesh is placed from the outside of the peritoneal cavity [8, 9]. However, it is impossible to diagnose the type of hernia or confirm repair after the application of the covering mesh because intraperitoneal observation cannot be performed. Furthermore, the operative time is longer than that of TAPP because of the increased difficulty in dissection and limited workspace [5, 7]. However, it is difficult to evaluate this factor because it is often dependent on the surgeon [10]. In our patient, intraperitoneal observation was necessary to check for intestinal nonischemia. In the case of incarcerated femoral hernia, the viability of the bowel segment is determined on the basis of color, peristalsis, and congestion. If observation of the intraperitoneal cavity reveals an intestinal incarceration in the femoral hernia sac, forceps are inserted from the same incision site, and the intestine is returned to the abdominal cavity without increasing the number of trocars used. Moreover, if intestinal ischemia is present, intestinal resection is required with a multichannel access port in the peritoneal space at the same umbilical incision.

However, securing a clear field of vision was difficult in our patient because of ileus. Hence, we performed iSTEP to use the advantages of TEP and TAPP. In iSTEP, intraperitoneal observation can diagnose the type of hernia and confirm mesh coverage in cases where the hernia extends not only to direct and indirect inguinal lesions but also to femoral and obturator lesions. Furthermore, it is possible to view the inguinal region without overlooking coexisting lesions, and extensive dissection, covering with the mesh, and dissection of the abdominal wall side are easy through intraperitoneal observation in combination with iSTEP [4]. Additionally, because it is a laparoendoscopic single-site surgery, all the procedures can be performed using the same incision. Hence, intraperitoneal operation and preperitoneal operation can easily be changed to obtain excellent cosmetic outcomes. Although the operative time is longer than that of conventional procedures and multiport laparoscopic surgery, the blood loss is equivalent, and the outcome is excellent with respect to postoperative complications [11–13]. Mesh repairs may result in no recurrence and may reduce the time until normal daily activities are resumed. However, nonmesh repair is less likely to cause infection. Therefore, the type of repair—mesh or nonmesh repair—should be carefully selected for the treatment of strangulated femoral hernia with bowel resection [14].

## Conclusion

We report a case of a patient who underwent successful iSTEP for strangulated femoral hernia who was at risk for intestinal ischemia after repositioning.

## Data Availability

Not applicable.

## Abbreviations

CT: Computed tomography
iSTEP: Single-incision totally extraperitoneal hernia repair with intraperitoneal inspection
TAPP: Transabdominal preperitoneal approach
TEP: Totally extraperitoneal hernia repair
